# Pulmonary Hypertension in Portugal: First Data from a Nationwide Registry

**DOI:** 10.1155/2013/489574

**Published:** 2013-10-21

**Authors:** Rui Baptista, José Meireles, Ana Agapito, Graça Castro, António Marinho da Silva, Teresa Shiang, Fabienne Gonçalves, Susana Robalo-Martins, António Nunes-Diogo, Abílio Reis

**Affiliations:** ^1^Department of Cardiology, Hospitais da Universidade de Coimbra, Centro Hospitalar e Universitário de Coimbra, Praceta Mota Pinto, 3000 Coimbra, Portugal; ^2^Institute for Biomedical Imaging and Life Sciences, Faculty of Medicine of University of Coimbra, Azinhaga de Santa Comba, 3000 Coimbra, Portugal; ^3^Department of Internal Medicine, Hospital Geral de Santo António, Centro Hospitalar do Porto, Largo Professor Abel Salazar, 4099 Porto, Portugal; ^4^Department of Cardiology, Hospital Santa Marta, Centro Hospitalar de Lisboa Central, Rua de Santa Marta, 1169 Lisboa, Portugal; ^5^Department of Pneumology, ó Hospital Santos Silva, Centro Hospitalar Gaia/Espinho, Rua Conceição Fernandes, 4434 Vila Nova de Gaia, Portugal; ^6^Department of Cardiology, Hospital de Santa Maria, Centro Hospitalar de Lisboa Norte, Avenida Professor Egas Moniz, 1649 Lisboa, Portugal

## Abstract

*Introduction*. Pulmonary arterial hypertension (PAH) is a rare disease that must be managed in specialized centers; therefore, the availability of epidemiological national data is critical. *Methods*. We conducted a prospective, observational, and multicenter registry with a joint collaboration from five centers from Portugal and included adult incident patients with PAH or chronic thromboembolic pulmonary hypertension (CTEPH). *Results*. Of the 79 patients enrolled in this study, 46 (58.2%) were classified as PAH and 33 patients (41.8%) as CTEPH. PAH patients had a mean age of 43.4 ± 16.4 years. Idiopathic PAH was the most common etiology (37%). At presentation, PAH patients had elevated right atrial pressure (RAP) (7.7 ± 5.9 mmHg) and mean pulmonary vascular resistance (11.4 ± 6.5 Wood units), with a low cardiac index (2.7 ± 1.1 L*·*min^−1^ *·*m^−2^); no patient was under selective pulmonary vasodilators; however, at follow-up, most patients were on single (50%), double (28%), or triple (9%) combination vasodilator therapy. One-year survival was 93.5%, similar to CTEPH patients (93.9%), that were older (60.0 ± 12.5 years) and had higher RAP (11.0 ± 5.2 mmHg, *P* = 0.015). *Conclusions*. We describe for the first time nationwide data on the diagnosis, management, and prognosis of PAH and CTEPH patients in Portugal. Clinical presentation and outcomes are comparable with those reported on other national registries.

## 1. Introduction

In the past few decades, the international scientific community has made great progresses in the understanding of the epidemiology, pathophysiology, and management of pulmonary arterial hypertension (PAH). It is a rare disease, malignant in character, and rapidly fatal, if not treated, with a median survival of 2.8 years in a historic cohort [1]. These progresses were accompanied by the development of drugs that target specific pathways in the pathophysiology of the disease [2]. Management in specialized centers and the use of pulmonary vasodilators lead to a significant impact on the survival and quality of life of PAH patients [3]. Unfortunately, survival rates are still unsatisfactory [4], signaling for the need of more effective treatments, which are under development [5].

Since the first consensus conference in 1973 [6], the classification of pulmonary hypertension (PH) has evolved, reflecting the ongoing understanding of the condition, and now it includes five groups with several subtypes [7]. Within group 1 PH, an idiopathic subgroup is maintained, highlighting that there is still a lot to understand about the pathogenesis of the disease. The diagnostic and therapeutic approach should be guided by national and international guidelines supported by scientific societies, and given the rarity and severity of the disease, its proper investigation and treatment should be performed in expert centers [8, 9].

Chronic thromboembolic pulmonary hypertension (CTEPH), classified as group 4 PH, has a different pathophysiology and treatment from other PH groups. Pulmonary endarterectomy (PEA) is a potentially curative procedure for CTEPH [10]. For those patients not eligible for surgery or those with persistent PH after PEA, specific treatment may ameliorate symptoms and enhance survival [9, 11].

The organization and publication of national and international registries are essential in the understanding of the epidemiology, etiology, and natural history of the different groups of PH [3]. Several groups have published data from their cohorts [12–17], although with different inclusion and exclusion criteria and methodologies [16]; to overcome these disparities, the creation of an international registry has been suggested [18]. Moreover, it is unclear if data from regional registries can be applied to other populations [13]. Data from national registries are not a surrogate for application in other countries and cannot be easily extrapolated due to demography, treatment availability, and other regional differences. Therefore, national registries from each region are paramount in the interpretation of the applicability of international recommendations, which are issued regardless of those differences. Our aim is to present data from a Portuguese registry of patients with group 1 and group 4 PH and to compare them with other published cohorts.

## 2. Population and Methods

We conducted a prospective, observational, and multicenter registry with a joint collaboration from five PH centers around Portugal. Although there are small differences between the institutions regarding patient follow-up, all of them follow similar protocols, according to the published national [8] and international [19] guidelines. Our study population consisted of adult incident PH patients referred to those centers for diagnostic and therapeutic evaluation, between 2008 and 2010. Data were collected by clinical file review by a physician, with supervision from the assistant PH physician, and were compiled in a dedicated software, specifically developed for the management of PH patients (PAHTool, Inovultus, Santa Maria da Feira, Portugal), creating a database and the backbone for a national registry. An informed consent was obtained from each patient, and the study protocol conforms to the ethical guidelines of the 1975 Declaration of Helsinki as reflected in a priori approval by the institution's human research committee. The centers' participation in this registry was voluntary, and the nationwide data collection was approved by the National Center for Data Protection. 

To enable comparisons with other published registries, we used strict inclusion and exclusion criteria. All patients had PAH confirmed by right heart catheterization (RHC), with a mean pulmonary arterial pressure (PAP) over 25 mmHg and a pulmonary wedge pressure (PCWP) equal or under 15 mmHg or a left ventricular end diastolic pressure (LVEDP) equal or under 15 mmHg. The date of diagnosis corresponds to the confirmation of PAH by RHC.

Studied data included demographic characteristics, clinical and laboratorial parameters, World Health Organization (WHO) functional class, haemodynamics, and conventional and specific vasodilator therapy usage and survival status. Vasoreactivity testing was performed when possible, using various institutional protocols. A one-year follow-up was conducted; no patients were lost to follow-up.

All results are expressed as the mean ± standard deviation or as the frequency. We used Kolmogorov-Smirnov for testing normality, Student's *t*-test for continuous variables, and *X* ^2^-test for categorical variables. Survival analysis was performed using the Kaplan-Meier method, and comparisons were made using the Log-Rank test. Values of *P* < 0.05 were considered to be significant. Statistical analysis was performed using SPSS 17.0 software package (IBM, New York, USA).

## 3. Results

**Figure 1:**
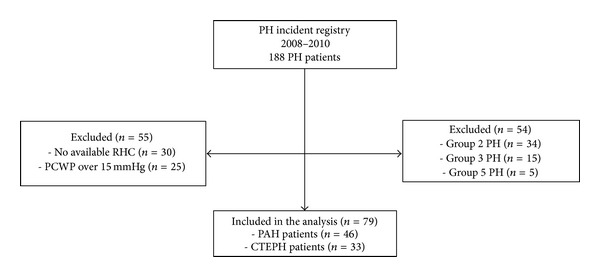
Patient selection flowchart.

Our registry originally included 188 PH patients (Figure 1). After exclusion of 79 patients from groups 2, 3, and 5 PH, 134 patients were left for analysis. Thirty patients were excluded as they did not have an available RHC. The final analysis included 79 patients. Of the 79 patients enrolled in this study, 46 (58.2%) patients were classified as PAH and 33 patients (41.8%) as CTEPH.

### 3.1. Demographics and Clinical Data

There was a clear preponderance of women among PAH patients, with a female/male patient ratio of 1.9 : 1. Mean age at diagnosis was 43.4 ± 16.4 years (range, 15 to 77 years) (Table 1). There was no difference among genders regarding age at first medical examination (*P* = 0.963). Among the 46 patients, 9.2% (*n* = 11) were <21 years old, 58.3% (*n* = 21) were 21 to 40 years old, 32.6% (*n* = 15) were 40 to 59 years old, and 17.4% (*n* = 8) were > 61 years old. Patients between 21 and 60 years of age accounted for 87% of all patients.

Idiopathic PAH was present in 17 (37%) patients, followed by connective tissue disease (CTD) (*n* = 12, 26%), congenital heart disease (CHD) (*n* = 10, 22%), portopulmonary hypertension (*n* = 5, 11%), familial (*n* = 1, 2%), and other etiologies (*n* = 1, 2%) (Table 2). At baseline, most patients presented in WHO class III or IV (71%); only one patient was in class I.

**Figure 2:**
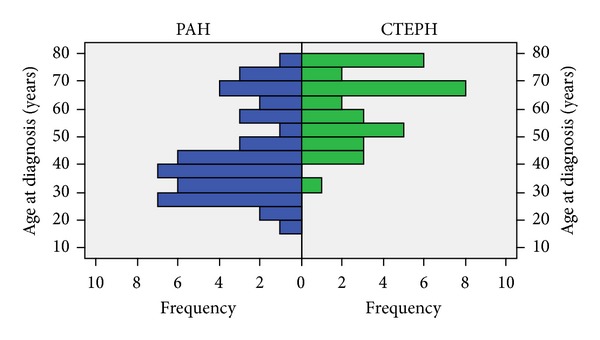
Distribution of age and gender.

CTEPH patients had a higher mean age at diagnosis (60.3 ± 12.5, *P* < 0.001) than group 1 PAH patients; a significant proportion of the population had more than 51 years at diagnosis (63.6%) (Figure 2). Both WHO class at presentation and the female/male ratio were similar to group 1 PAH patients.

### 3.2. Hemodynamics

RHC was performed in all patients at the initial examination (Table 1). Baseline data shows that in group 1 PAH patients, mean RAP was 7.7 ± 5.9 mmHg, mean PAP was 50.6 ± 17.9 mmHg, and mean PCWP was 9.5 ± 3.5 mmHg; PVR was 11.4 ± 6.5 Wood units. Mean cardiac output (CO) was 4.5 ± 1.8 L·min^−1^, and mean cardiac index (CI) was 2.7 ± 1.1 L·min^−1^·m^−2^. Cardiac output was more elevated in WHO class I/II than in the WHO class III or IV patients, but it did not reach statistical significance. Conversely, PVR was higher in patients in WHO class III/IV than patients in WHO class I/II (Table 3). Vasoreactivity testing was performed in 29 (63.0%) patients with various protocols; 6 patients (21%) had a positive test.

Regarding CTEPH, the only hemodynamic parameter at the time of diagnostic RHC that was significantly different from PAH was the mean RAP (11.0 ± 5.2 mmHg, *P* = 0.015), that was significantly higher.

### 3.3. Treatment

Drug therapy at study inclusion is shown in Table 4. At baseline, all PAH patients were treated only with conventional therapy. Diuretics were used by 15 patients (32.6%), followed by oxygen in 9 patients (19.6%) and digoxin in 7 patients (15.2%). At follow-up, 42 patients were treated with advanced PAH therapies and 40 with pulmonary vasodilators, and two patients were enrolled in randomized controlled trials (RCT) (Table 5). Most patients were medicated with endothelin receptor antagonists (*n* = 33), followed by phosphodiesterase inhibitors (*n* = 26) and prostanoids (*n* = 4). Thirteen patients (28%) were under double combination therapy and 4 (9%) patients under triple combination therapy.

No differences were found regarding baseline treatment modalities among PAH and CTEPH patients. However, during follow-up, targeted therapies were begun in 67% of CTEPH patients, and 5 patients (15.2%) had a PEA. Combination therapy was offered to 9 CTEPH patients during the follow-up period. Endothelin receptor antagonists were used in 17 patients, followed by sildenafil in 13 patients and prostanoids in 2 patients. One patient was enrolled in a RCT. 

### 3.4. One-Year Survival Analysis

**Figure 3:**
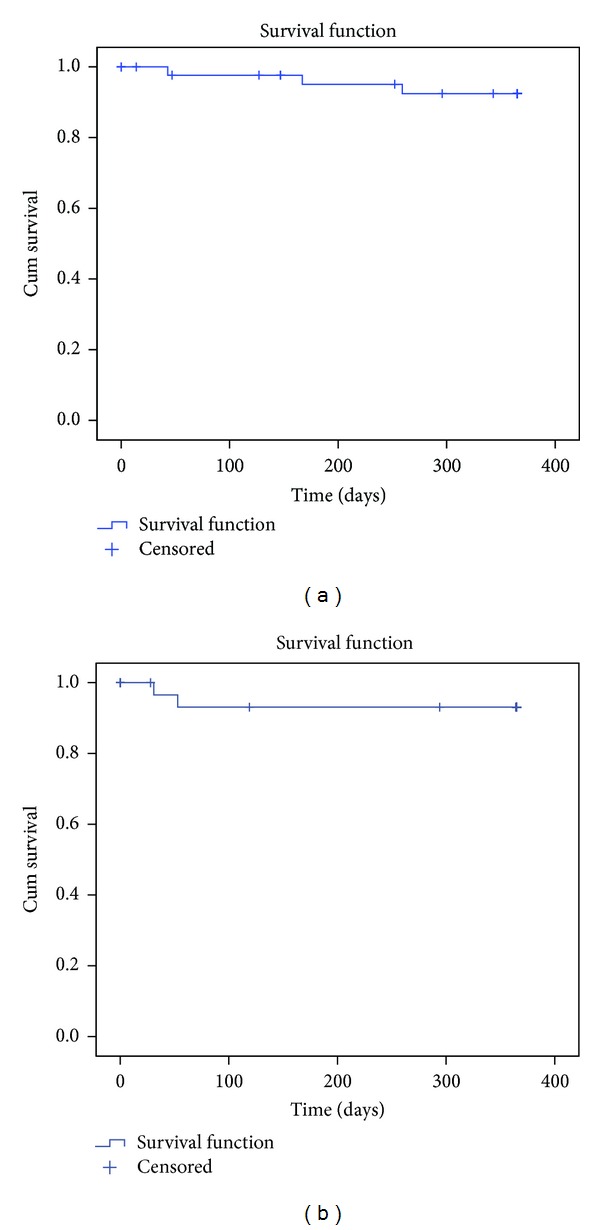
One-year survival in pulmonary arterial hypertension (a) and chronic thromboembolic pulmonary hypertension (b) patients.

Survival data was available for all patients (Figure 3). One year after the diagnostic RHC, 5 patients were deceased. The Kaplan-Meier survival estimates for patients with PAH and CTEPH at 1 year were 93.5% and 93.9%, respectively (Log-rank *P* = 0.709). Unoperated CTEPH patients had a one-year survival rate of 92.9%, whereas all patients that underwent PEA survived.

### 3.5. Comparison with the Cohort of Group 1 PAH Patients without Available Baseline RHC

The original database included 20 patients classified as PAH but without an available baseline RHC; therefore, they were not included in the analysis. This may have been due to the incomplete filling of the database fields and thus not necessarily reflecting the absence of RHC. Comparing with included PAH patients, we found no significant differences regarding gender, age, or WHO class at presentation of these patients. Although not reaching statistical significance, there was a trend for a higher proportion of patients with CHD-associated PAH in the group of patients that did not had a RHC. No survival differences were found among the two groups.

### 3.6. Estimated Incidence of PAH and CTEPH

Although limited by the voluntary collection of data and by the selective inclusion and exclusion criteria, we identified 46 patients with incident group 1 PAH and 33 patients with CTEPH during the 3-year follow-up period. For a population of 10 million inhabitants in Portugal, we calculated a conservative estimation of group 1and group 4 PH annual incidence of at least 1.5 and 1.1 patients per million, respectively. However, if we include patients with a clinical diagnosis of group 1 PAH but without an available RHC, our incidence would rise to 2.2 per million per year.

## 4. Discussion

The present study summarizes data representative of the Portuguese PH cohort. With the combined effort of five treatment centers, we were able for the first time to collect nationwide data on the diagnosis, management, and clinical course of PAH and CTEPH in Portugal.

To analyze a homogeneous population and to enable comparisons with other published cohorts [12, 17, 20], we followed strict inclusion and exclusion criteria based on current guidelines [9]. We focused only on PAH and CTEPH patients, as the prevalence and clinical characteristics of group 2 and group 3 PH varied widely among the five PH centers. Additionally, we included only incident cases to remove survivor bias from our study and to permit an approximate calculation of annual incidence, as prevalent cases correspond mainly to survivors [12].

In our PAH population, age at diagnosis was lower than in the REVEAL [20] and French [12] cohorts but higher than in the NIH registry [21]. This may be due to the fact that one-fifth of our patients had CHD-associated PAH; these patients were excluded from the French registry [12] but not from the REVEAL cohort [20]. Other important aspect is that almost 20% of PAH patients were over 60 years, a finding that is being increasingly recognized in contemporary registries. CTEPH patients were older, with a mean age at diagnosis of 60 years, similar to the one found in the Swiss cohort [22] and the randomized clinical trial [23]. Interestingly, our CTEPH population was significantly older than a published cohort of patients developing CTEPH after an acute pulmonary embolism [24]. This fact may warrant further investigation and may signalize a different epidemiology of postpulmonary embolism CTEPH.

Possibly due to the fact that we have no patients with PAH secondary to anorexigens, a group almost exclusively formed by women [25], the proportion of female patients (65%) in our PAH cohort was lower than that in most published reports [26].

Idiopathic PAH was the most frequent subgroup of PAH, with a proportion of 37%, similar to the French cohort (41% in the incident cohort) [12] but lower than in the REVEAL cohort (47%) [20]. CTD-PAH was the second most common cause, with 26%, a number that is higher than that reported on the French (18%) [12] but similar to the REVEAL cohort (24%) [20]. Systemic sclerosis is the leading cause of CTD-PAH, with 8% of patients developing this dismal prognostic finding in the course of their disease [27]; echocardiographic screening may be of value and has a grade IIb C recommendation on the current ESC guidelines [9].

As expected, patients with CHD-PAH were frequent in our series (22%), a significantly higher proportion than in the French and REVEAL cohorts [12, 20]. This may be the result of the poor access of CHD patients to corrective heart surgery in the appropriate age; however, CHD patients are also clearly underrepresented in other epidemiological series, as in the French cohort due to health organization issues [12]. Portopulmonary hypertension had a similar incidence to the French series.

PAH baseline haemodynamics was similar to those from the NIH, REVEAL French Comparison Cohort (FCC), and French registry. The mean PAP was 51 mmHg, being essentially the same of the French registry (55 mmHg) [12] and the REVEAL FCC (51 mmHg) [26] and slightly lower than the NIH cohort (60 mmHg) [1]. Mean RAP at diagnosis was 8 mmHg, the same as the French (8 mmHg) and REVEAL FCC registry (8 mmHg). 

Most PAH patients presented to the referral center with symptoms of advanced heart failure. In 71% of cases, they were in WHO class III or IV, similarly to the REVEAL FCC (73%) [26] and the French registry (75%) [12]. This number is even more dramatic as it is similar to the one reported on the 20-year-old NIH cohort (71%) [21]. The combination of high RAP with an advanced functional class on presentation signalizes that more effort is needed for early identification and referral of patients to expert centers, as there is evidence that treating patients in WHO class II has a positive impact on patients' outcomes [28].

CTEPH patients had similar haemodynamics compared to PAH, except for mean RAP, which was significantly higher. This finding that was not reproduced in the Swiss cohort [22], is higher than that reported in the BENEFIT study [23] and in the Cambridge cohort [29]. Higher right ventricular filling pressures were accompanied by a higher number of patients being referred to WHO class III or IV (78%), although similar to the proportion reported in the literature [29]. This may indicate late identification and the referral of these potentially curable patients.

Although having high WHO functional class at presentation, most PAH patients were not treated with diuretics when referred to the expert centers. The same was true regarding CTEPH patients; however, there was a trend for more intense anticongestive medication in this group. Our results are comparable to those from the Swiss registry [22]. Late referral to specialized centers may be in part due to the lack of an nationwide reference network and availability of oral vasodilator drugs for PAH treatment, as less specialized centers may delay transfer patients to expert centers [12]. In our population, no patient was treated with specific pulmonary vasodilators before being referred to the specialized centers.

Vasoreactivity testing was held in 29 of 46 PAH patients (63%) and was positive in 6 patients (21%). This value is significantly higher than that reported by the French cohort (10.3%) [12] but similar to the Swiss registry (20%) [17], although the latter included 8 CTEPH patients. Selection bias, differences in the definition of acute responders and in the treatment protocols used nationwide, may be responsible for the inconsistencies. Publication of national guidelines may help to standardize the care for this group of patients.

The progress in prognosis is inseparable from the advances in pulmonary vasodilator therapy. There are three classes of selective vasodilator drugs that target three critical pathways in PAH (prostacyclin, nitric oxide, and endothelin-1) [2], all being available in Portugal. All of them have their efficacy demonstrated in several randomized controlled trials regarding functional capacity, exercise tolerance, haemodynamics, and other endpoints [30]. Moreover, a recent meta-analysis confirmed the impact of pulmonary vasodilators on short-term survival [11]. Overall, in our cohort the one-year survival for PAH patients was 93.5%, similar to the REVEAL (91.0%) [20] and the Swiss cohort (89.0%) [22] but higher than the French cohort (85.7%) [12]. The differences may be accounted by the small number of events in our cohort (5 deaths) and the relative higher proportion of patients with CHD-associated PAH (21.7%) compared with the REVEAL (11.8%), Swiss (approximately 14.3%), and French cohorts (0%) [12, 17, 20]. These patients clearly have a better prognosis [31], and thus they may have contributed to the positive survival results. 

CTEPH patients had a similar one-year survival (93.9%); only five patients underwent PEA, a potentially curative procedure that probably had impact on prognosis, as no patient died on follow-up. All CTEPH patients are assessed by the local medical PH team, and the potential surgical candidates are discussed directly with the foreign referral center, as the procedure was not routinely performed in our country. Not operated patients had a survival of 92.9%. For these patients and for those with residual PH after PEA, pulmonary vasodilator drug therapy is a class IIb C recommendation in the ESC guidelines [9]. In our cohort, two thirds of CTEPH patients started specific therapy, a number that is comparable to other series [29]. Interestingly, the one-year survival of the not operated CTEPH patients was similar to that recently reported in the literature (96%) [32].

Our estimated PAH annual incidence between 1.5 and 2.2 cases per million inhabitants is in line with the published incidences in other countries: Belgium (1.7 per million) [33], Israel (1.4 per million) [34], France (2.4 per million) [12], Switzerland (2.4 per million) [17], and USA (2.0 per million) [26]. The wide range between the most conservative estimate and the higher value is also observed in other series, as in France, where there are very high regional differences in PAH prevalence, ranging from 5 to 25 per million inhabitants per year [12]. CTEPH had, in our population, an estimated incidence of 1.1 cases per million, a number similar to that reported in the United Kingdom in 2001 (1.02 cases per million) but lower than that in 2005 (1.75 cases per million) [29].

Our study has several limitations. First, although we included data from five expert PH centers in Portugal, there are patients followed in other hospitals across the country. This had impact on incidence calculations, namely, an underestimation of values, both in PAH and CTEPH. Second, we used strict inclusion and exclusion criteria for the recruitment of patients in this registry to ensure a homogenous population. This has caused the exclusion of all patients without a RHC on the databases, whether or not there was one available on the clinical files. However, as our mean hemodynamic values, demographics, and functional class data are in line with those published on the literature, we believe that our population is representative and has external validity. Thirdly, we decided not to include groups 2, 3, and 5 patients, as there were significant differences among centers regarding the clinical characteristics of these patients. A careful analysis was made, but avoiding confounding factors that are frequent in observational studies may have been impossible [35]. 

In conclusion, the present unique study reports for the first time data on the epidemiology, clinical characteristics, and prognosis of PAH and CTEPH patients in Portugal. We conclude that overall PAH incidence is similar to that reported in other European series, but patients are still being diagnosed late in the course of their disease. We also report that CHD-PAH is an important etiology in our country and may need special attention. The one-year survival analysis of our incident cohort exceeds 93%, a value that reflects access to contemporary treatment of PAH, being a strong incentive to the continuous work being developed by all the community members involved in this disease. We demonstrated that a combined and organized registry is possible and is a useful tool to obtain quality data for clinical decision-making that compares well with data from other registries. Our findings encourage the amplification and maintenance of a nationwide registry by the combined effort of all the physicians caring these patients, aiming for a better care and prognosis of PH patients.

## Figures and Tables

**Table 1:** Demographic, clinical, and hemodynamic characteristics of pulmonary arterial hypertension (PAH) and chronic thromboembolic pulmonary hypertension (CTEPH) incident patients at baseline.

	Total (*n* = 79)	PAH (*n* = 46)	CTEPH (*n* = 33)	*P* value
Age (years)	50.5 ± 17.0	43.4 ± 16.4	60.3 ± 12.5	<0.001
Female gender, *n* (%)	53 (67.1%)	30 (65.2%)	23 (69.7%)	0.676
Six-minute test walking distance (m)	351.3 ± 137.4	370.8 ± 140.1	320.4 ± 132.9	0.327
Functional class, *n* (%)				
I	1 (1%)	1 (2%)	0 (0%)	0.565
II	17 (25%)	11 (27%)	6 (21%)	
III	34 (49%)	21 (51%)	13 (46%)	
IV	17 (25%)	8 (20%)	9 (32%)	

Hemodynamic data				

Right atrial pressure (mmHg)	9.1 ± 5.8	7.7 ± 5.9	11.0 ± 5.2	0.015
Mean pulmonary artery pressure (mmHg)	49.1 ± 15.1	50.6 ± 17.9	47.1 ± 9.9	0.313
Cardiac output (L·min^−1^)	4.4 ± 1.9	4.5 ± 1.8	4.3 ± 2	0.778
Cardiac index (L·min^−1^·m^−2^)	2.6 ± 1.1	2.7 ± 1.1	2.5 ± 1.1	0.406
Pulmonary capillary wedge pressure (mmHg)	9.7 ± 3.3	9.5 ± 3.5	9.9 ± 3.1	0.587
Pulmonary vascular resistance (Wood units)	11.1 ± 6.4	11.4 ± 6.5	10.8 ± 6.3	0.729

**Table 2:** Clinical and hemodynamic data stratified by pulmonary arterial hypertension subgroup.

Subgroup	*N* (%)	Female (%)	Age (years)	WHO I/II (%)	6MWT (meters)	RAP (mmHg)	mPAP (mmHg)	CO (L·min^−1^)	PCW (mmHg)	PVR (WU)
Idiopathic	17 (37.0)	70.6	37.5 ± 12.9	31.3	405 ± 121	11 ± 6	53 ± 15	4.2 ± 1.5	10.7 ± 3.3	11.7 ± 5.6
CTD	12 (26.1)	75.0	56.8 ± 12.4	27.3	275 ± 127	6 ± 6	39 ± 11	4.9 ± 1.8	7.6 ± 3.2	8.7 ± 7
CHD	10 (21.7)	50.0	37.7 ± 15	22.2	351 ± 171	6 ± 5	60 ± 27	4.6 ± 2.7	10.7 ± 3.7	13.9 ± 8.9
PortPulm	5 (10.9)	60.0	51.2 ± 18.3	33.3	n/d	7 ± 5	51 ± 11	4 ± 1.2	8.6 ± 2.9	11.1 ± 3.3

Total	44 (100.0)*	65.2	43.4 ± 16.4	29.3	371 ± 140	8 ± 6	51 ± 18	4.5 ± 1.8	9.5 ± 3.5	11.4 ± 6.5

CTD: connective tissue disease; CHD: congenital heart disease; PortPulm: portopulmonary. WHO: World Health Organization; 6MWT: six-minute walking test distance; RAP: right atrial pressure; mPAPA: mean pulmonary artery pressure; CO: cardiac output; PCWP: pulmonary capillary wedge pressure; PVR: pulmonary vascular resistance; WU: wood units.

*Heritable PAH (*n* = 1) and other etiologies PAH (*n* = 1) were not reported as there was one case of each in the cohort.

**Table 3:** Hemodynamic characteristics stratified by NYHA class of pulmonary arterial hypertension incident patients.

(*n* = 46)	NYHA I/II	NYHA III	NYHA IV	*P* value
Six-minute walk test distance (m)	436 ± 147	356 ± 106	236 ± 128	0.094
Female gender	67.8%	71.4%	37.5%	0.229
Right atrial pressure (mmHg)	8 ± 6	8 ± 6	7 ± 7	0.854
Mean pulmonary artery pressure (mmHg)	54 ± 26	49 ± 11	45 ± 13	0.492
Pulmonary capillary wedge pressure (mmHg)	9 ± 3	10 ± 4	7 ± 3	0.092
Cardiac output (L·min^−1^)	5.4 ± 0.9	4.6 ± 2.1	3.2 ± 1.6	0.097
Pulmonary vascular resistance (Wood units)	9.1 ± 4.6	10.6 ± 5.6	15.3 ± 6.1	0.123

**Table 4:** Conventional therapies at baseline and follow-up of pulmonary arterial hypertension (PAH) and chronic thromboembolic pulmonary hypertension (CTEPH) patients.

	Total		PAH		CTEPH	
	Baseline	Follow-up	Baseline	Follow-up	Baseline	Follow-up
Diuretics	33 (41.8%)	40 (65.6%)	15 (32.6%)	17 (60.7%)	18 (54.5%)	23 (69.7%)
Digoxin	13 (16.5%)	18 (2.8%)	7 (15.2%)	11 (23.9%)	6 (18.2%)	7 (21.2%)
Oxygen	21 (26.6%)	25 (31.6%)	9 (19.6%)	12 (26.1%)	12 (36.4%)	13 (39.4%)
Warfarin	34 (43.0%)	59 (74.7%)*	10 (21.7%)	28 (60.9%)*	24 (72.7%)	31 (93.9%)*

*P < 0.001 versus baseline.

**Table 5:** Pulmonary vasodilator therapies at follow-up of pulmonary arterial hypertension (PAH) and chronic thromboembolic pulmonary hypertension (CTEPH) patients.

	PAH	CTEPH
No advanced therapies	2%	33%
Single therapy	50%	36%
Double combination therapy	28%	15%
Triple combination therapy	9%	7%
Calcium channel blockers	7%	0%
Randomized controlled trial drug	4%	3%
